# Investigating the Action Mechanism of Titanium in Alumina–Magnesia Castables by Adding Different Ti-Bearing Compounds

**DOI:** 10.3390/ma15030793

**Published:** 2022-01-21

**Authors:** Hai Tang, Yuhao Zhou, Wenjie Yuan

**Affiliations:** 1 The State Key Laboratory of Refractories and Metallurgy, Wuhan University of Science and Technology, Wuhan 430081, China; tanghaiwust@sina.com (H.T.); heltlong@gmail.com (Y.Z.); 2 National-Provincial Joint Engineering Research Center of High Temperature Materials and Lining Technology, Wuhan University of Science and Technology, Wuhan 430081, China

**Keywords:** Ti-bearing compounds, CA_6_, spinel, castables

## Abstract

To investigate the action mechanism of titanium, the effects of different Ti-bearing compounds, including CaTiO_3_, MgTiO_3_, and nano-TiO_2_, on the properties of alumina–magnesia castables were studied. By analyzing the phase compositions, microstructures, and physical and mechanical properties of the castables, it was demonstrated that an intermediate product, CaTiO_3_, was first generated. This was then consumed by solid-solution reactions, and titanium was involved in the liquid formation as the temperature increased. The solid-solution reaction of CA_6_ (CaAl_12_O_19_) was more prominent due to the incorporation of more titanium in the crystal lattice of CA_6_ instead of spinel (MgAl_2_O_4_). Moreover, the liquid formation was strongly promoted when more titanium accompanied the calcium, which finally accelerated the densification and improved the strengths of alumina–magnesia castables. On the whole, castables with CaTiO_3_ addition presented higher bulk density and excellent strength after the heat treatment. Besides, the castables with 2 wt.% CaTiO_3_ contents were estimated to possess greater thermal shock resistance.

## 1. Introduction

Alumina-magnesia castables served at high temperatures mainly comprise alumina, spinel, and CA_6_. The presence of spinel can improve the slag corrosion and erosion resistances of castables due to its high chemical resistance and mechanical properties [1,2,3,4]. Ko [5] reported that alumina-magnesia castables had better slag resistance than alumina-spinel castables, because the in-situ spinel possessed smaller grain sizes. Furthermore, CA_6_ formed by the reaction between calcium aluminate cement and reactive alumina powders resulted in excellent mechanical strengths of the castables owing to the bridging and deflection mechanism of platelet CA_6_ [6]. The high stability in the reducing atmosphere and low solubility of CA_6_ in slag also allow it to be in contact with molten iron and steel [7]. However, the formation of CA_6_ and spinel leads to volume expansions of 3.01 and 8%, respectively, which would result in the spalling of castables at elevated temperatures [8]. Thus, mineralizers, such as TiO_2_, B_2_O_3_, ZrO_2_, and rare earth oxides, have been introduced into this system to accelerate the densification of castables [9,10].

Among these additives, TiO_2_ is regarded as one of the most effective multifunctional mineralizers, which can control the expansion behavior of alumina-magnesia castables and speed up the formation of CA_6_ and spinel [11]. The densification of castables can be accelerated to a great extent due to the larger amount liquid phase during low viscosity forming when TiO_2_ is incorporated [12]. In a previous study, it was found that the apparent activation energy of spinel formation varied with the content of TiO_2_ in the same system [13]. Furthermore, the morphology of CA_6_ could be influenced by the addition of nano-TiO_2_ [14]. TiO_2_ also functioned as a nucleation agent, promoting the recrystallization of secondary spinel from the liquid phase [15].

Although extensive investigations have found that TiO_2_ has a significant influence on the phase evolution of alumina-magnesia castables, few studies have focused the differential impacts of titanium on the formation of spinel and CA_6_. Therefore, it is necessary to further evaluate the contribution of titanium on the complex phase evolution of alumina-magnesia castables. Imbalanced distributions of titanium were designed using three kinds of Ti-bearing compounds (CaTiO_3_, MgTiO_3_, and nano-TiO_2_) in this work. It was assumed that more titanium was located in the region where CA_6_ and spinel formed in the samples with CaTiO_3_ and MgTiO_3_ addition, respectively. For the reference group (samples with nano-TiO_2_ addition), titanium was considered to be evenly distributed in the starting raw materials of the castables. The local compositions were adjusted, and the different effects of titanium on the formation of spinel and CA_6_ were investigated.

## 2. Materials and Methods

The formulations of the alumina-magnesia castables are listed in Table 1. Coarse tabular alumina (Almatis, Germany) was used as aggregates and fine particles served as the matrix of the castables. Reactive alumina (CL370, Almatis, Germany) and calcined magnesia (Dashiqiao, China) were designed to form in situ spinel at high temperatures. Calcium aluminate cement (Secar71, Kerneos, France) acted as the binder of the castables and a source for the CA_6_ formation. The added mineralizers included nano-TiO_2_ (Aladdin, Shanghai, China, 99.8 wt.% of purity), CaTiO_3_ (Aladdin, Shanghai, China, 99.5 wt.% of purity), and MgTiO_3_ (Aladdin, Shanghai, China, 99 wt.% of purity). Under the action of silica fume (951U, Elkem, Norway) and water reducer (FS60, BASF, Ludwigshafen, Germany), the amount of distilled water added was about 4.3 wt.%. The chemical compositions of raw materials are shown in Table 2. Samples with Ti-bearing compounds were divided into two groups, and the amounts of CaTiO_3_ and MgTiO_3_ were calculated to be 1 and 2 wt.% TiO_2_, respectively. Moreover, the contents of calcined magnesia and calcium aluminate cement were adjusted to ensure that the proportions of calcium and magnesium in the same batch did not vary.

The castables were mixed for 4 min in a rheometer with 4.3 wt.% distilled water. After mixing, the samples were cast in rectangle molds (150 × 25 × 25 mm) and then cured at 25 °C for 24 h in a climatic chamber with a relative humidity of 100%. After drying at 110 °C for 24 h, all the samples were calcined at 1150 °C, 1250 °C, 1350 °C and 1450 °C for 3 h, respectively. The permanent linear change (PLC) of the specimens was obtained by calculating the variation of the dimensions after cooling. The apparent porosity and bulk density of samples were measured by the Archimedes technique following the GB/T 2997-2000 standard [16]. The cold modulus of rupture was measured by a three-point bending test following GB/T 3001-2007 [17]. The elastic modulus of samples was measured by Elastic Modulus & Damping System (RFDA, HTVP1600, IMCE, Genk, Belgium). The phase compositions of the castables were characterized by X-ray diffraction (XRD, X’pert Pro MPD, Philips, Almelo, The Netherlands), and the relative results were analyzed based on the reference intensity ratio (RIR) method using the X’pert Highscore 2.0 Plus software. The microstructures of the castables were characterized by scanning electron microscopy (SEM, JEOL JSM-6610, JEOL Ltd., Tokyo, Japan). The chemical compositions and elemental distributions were analyzed using energy-dispersive X-ray spectroscopy (EDS, Bruker QUANTAX200-30, Karlsruhe, Germany).

## 3. Results

### 3.1. Phase Composition

**Figure 1:**
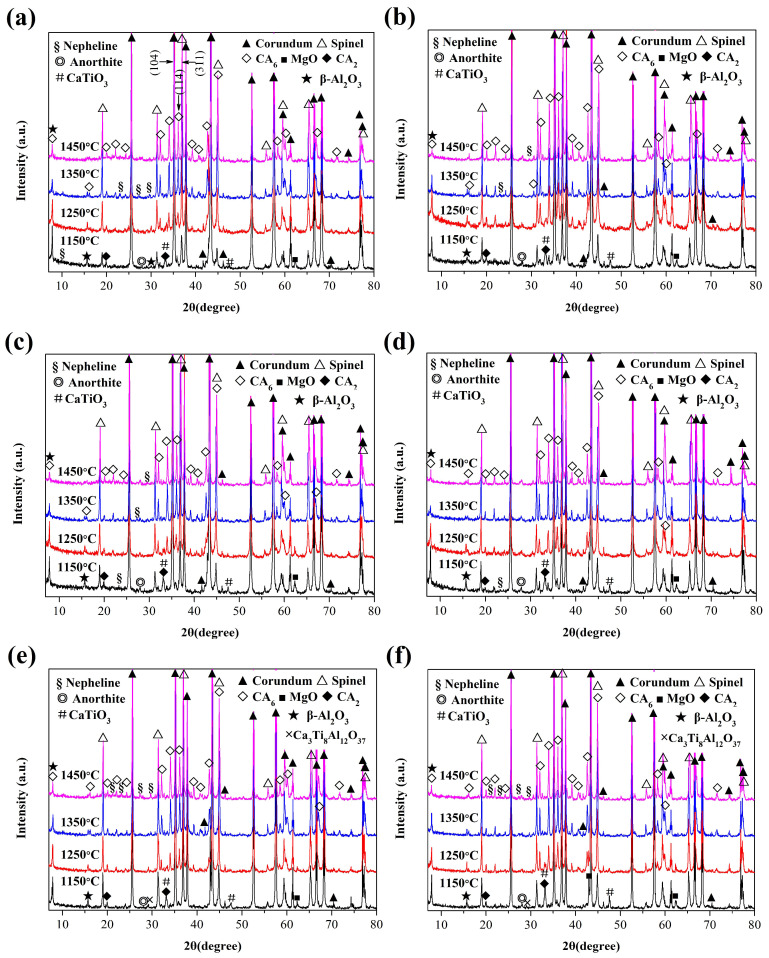
XRD patterns of the castables with the addition of different Ti-bearing compounds after calcination at different temperatures: (**a**) NT1, (**b**) NT2, (**c**) CT1, (**d**) CT2, (**e**) MT1, and (**f**) MT2.

XRD patterns of the castables containing different Ti-bearing compounds are presented in Figure 1. Spinel and CA_6_ formed at 1150 and 1250 °C in all the specimens. As reported in a previous study, the formation temperature of CA_6_ was up to 1400 °C in alumina-magnesia castables without mineralizer addition [12]. This demonstrated that the formation of CA_6_ was accelerated to a great extent by the introduction of Ti-bearing compounds. In addition, other minor phases, including β-Al_2_O_3_ (NaAl_11_O_17_), CA_2_ (CaAl_4_O_7_), MgO, and CaTiO_3_, as well as multicomponent products, such as nepheline (NaAlSiO_4_), anorthite (CaAl_2_Si_2_O_8_), and Ca_3_Ti_8_Al_12_O_37_, were also detected in the samples calcined at 1150 °C. It is well known that β-Al_2_O_3_ is derived from commercial alumina, and the liquid phase is usually first generated between β-Al_2_O_3_ and silica [18]. More β-Al_2_O_3_ participated in the formation of the liquid as the temperature increased, which resulted in a gradual decrease in the intensity of the diffraction peak. Calcium existed in the form of CA_2_, CaTiO_3_, and anorthite at a relatively low temperature (1150 °C) and then gradually transformed to CA_6_ as the temperature increased. MgO was gradually consumed simultaneously, and more spinel was generated with the increase in temperature.

CaTiO_3_ was detected instead of MgTiO_3_ (trace Ca_3_Ti_8_Al_12_O_37_ was also found for samples MT1 and MT2) in all the samples calcined at 1150 °C. This indicated that nano-TiO_2_ and MgTiO_3_ reacted with calcium aluminate to form CaTiO_3_ at this temperature. As the temperature increased further, Ti-bearing compounds gradually took part in the complex phase evolution of the matrix. Titanium could be incorporated into the crystal lattice of spinel and CA_6_, forming a solid solution [13]. Additionally, Ti-bearing compounds could participate in the formation of liquid [12]. These factors could account for the disappearance of Ti-bearing compounds, especially for CaTiO_3_ with calcination temperatures above 1150 °C.

**Figure 2:**
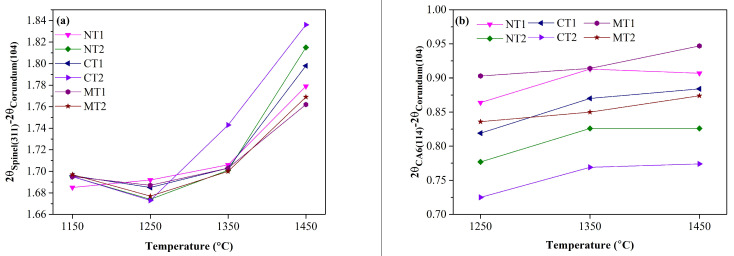
The shift of diffraction angle of spinel and CA_6_ in samples calcined at different temperatures: (**a**) spinel and (**b**) CA_6_.

Because the formation of a solid solution would cause a change of the lattice parameters, the variations of the diffraction peak positions for spinel and CA_6_ were compared to evaluate the doping level of titanium, as presented in Figure 2. The (104) plane of corundum was set as the base plane, and the selected crystal planes of spinel and CA_6_ were (311) and (114), respectively. Basically, the diffraction peaks of spinel gradually shifted to higher angles as the temperature increased, as presented in Figure 2a. This could be explained by the formation of Al-rich spinel caused by the aluminum substitution on the magnesium sites [19]. Because the radius of Al (0.143 nm) was less than that of Mg (0.160 nm) [20], the substitution of magnesium with aluminum could reduce the lattice parameter of spinel followed by an increase in the diffraction angle (2θ).

Additionally, the shift of the diffraction peak for spinel and CA_6_ depended on different Ti-bearing compounds. It has been demonstrated that the doping of titanium into the spinel and CA_6_ crystal lattices could cause a slight increase in the lattice constants [12,21]. The diffraction peaks of spinel in the samples with MgTiO_3_ addition calcined at 1450 °C were located at somewhat lower angles than the other peaks. Similarly, the diffraction angle of the CA_6_ peak in specimens with CaTiO_3_ addition was smaller than those of the other peaks. Moreover, the differences of the diffraction peaks of CA_6_ between the specimens were more significant compared with the differences of the peaks of the spinel phase. This demonstrated that the solid-solution reaction of CA_6_ strongly depended on Ti-bearing compounds.

**Figure 3:**
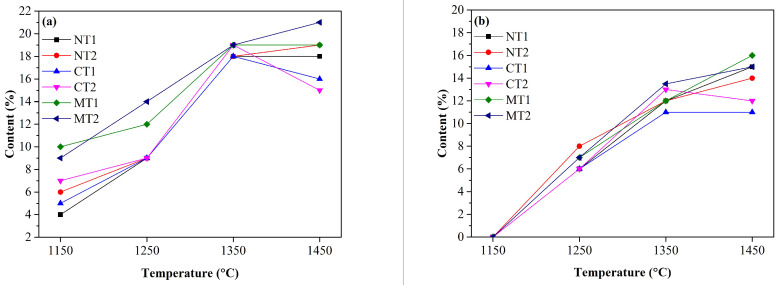
Spinel (**a**) and CA_6_ (**b**) contents in alumina-magnesia refractory castables containing Ti-bearing compounds as a function of the calcination temperature.

CA_6_ and spinel contents in the specimens were calculated by the RIR method, as presented in Figure 3. The formation of spinel in the samples with MgTiO_3_ addition (MT1 and MT2) was accelerated at 1150 and 1350 °C, as shown in Figure 3a. When the calcination temperature reached 1350 °C, the differences of spinel and CA_6_ contents between the samples were limited. Nevertheless, MgTiO_3_ addition favored the formation of spinel and CA_6_ at 1450 °C. In contrast, the amounts of spinel and CA_6_ in the samples with CaTiO_3_ addition were significantly lower than those of the other samples calcined at this temperature, which indicated that the formation of liquid consumed MgO, Al_2_O_3_, and CaO in the castables [18]. Thus, the decline of CA_6_ and spinel contents was likely related to greater liquid formation, which is discussed in the following section.

### 3.2. Microstructure

**Figure 4:**
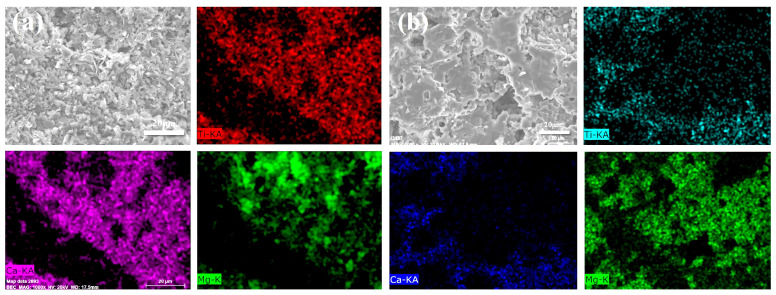
Titanium, calcium, and magnesium distribution in specimens after calcination at 1450 °C for 3 h: (**a**) MT2 and (**b**) CT2.

As illustrated above, Ti-bearing compounds participated in the complex reactions at higher temperatures, followed by the disappearance of diffraction peaks, as shown in Figure 1. The distributions of titanium in samples CT2 and MT2 calcined at 1450 °C were characterized by SEM/EDS mapping analysis (Figure 4). Titanium was mainly identified in the region where calcium was located rather than magnesium when MgTiO_3_ or CaTiO_3_ was added to the castables. Calcium and magnesium mainly appeared in the form of CA_6_ and spinel in the samples calcined at 1450 °C, as shown by the XRD patterns. Therefore, the incorporation of titanium in the CA_6_ crystal lattice was more prominent. It was reported that large amounts of titanium can dissolve into the structure of CA_6_, and the TiO_2_ concentration in hibonite can be as high as 10 wt.% [22]. In contrast, the occupation of Ti^4+^ at Al^3+^ sites in the spinel crystal lattice requires to overcome a higher energy barrier [23]. Thus, it was easier for titanium to form a solid solution with CA_6_ rather than spinel. Additionally, the transformation of MgTiO_3_ to CaTiO_3_ at a low temperature of 1150 °C (as discussed at the phase compositions) also resulted in the preferential distribution of titanium with calcium. These factors explained why the titanium was almost always detected in the area where calcium existed in the CA_6_ region.

**Figure 5:**
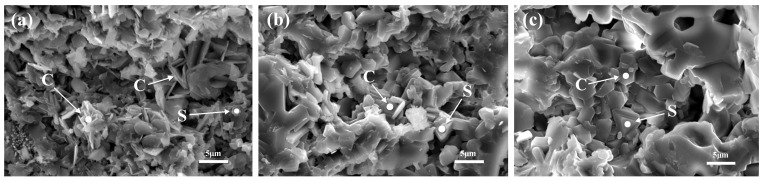
Microstructure of CA_6_ (C) and spinel (S) in samples calcined at 1450 °C for 3 h: (**a**) MT2, (**b**) NT2, and (**c**) CT2.

The microstructures of CA_6_ and spinel observed by SEM are shown in Figure 5. Thinner, flaky CA_6_ was stacked in the matrix of sample MT2 (shown in Figure 5a). CA_6_ in samples NT2 and CT2 exhibited thicker geometric characteristics (Figure 5b,c). The grain sizes of spinel in samples NT2 and CT2 were significantly larger than those in samples MT2. To reveal the mechanism that caused the morphological changes of CA_6_ and spinel, the chemical compositions of CA_6_ and spinel characterized by EDS analysis are listed in Table 3 (the EDS location is marked with a white point in Figure 5). The proportions of titanium in CA_6_ of samples NT2 and CT2 were significantly higher than that of sample MT2. Some studies found that the substitution of aluminum by titanium can greatly promote the mass transfer and grain growth along vertical axes (c-axes) [24]. This indicates that more titanium replaced aluminum in the CA_6_ structure, and the thicker, flaky CA_6_ was generated in the matrix of the castables. In addition, the substitution of aluminum by titanium can lead to numerous vacancies, which might promote the solid solution formation in spinel and CA_6_. Therefore, the doping amount of magnesium increased with more titanium doping into the CA_6_ crystal lattice. Only small amounts of titanium and calcium were incorporated into the crystal lattice of spinel (Table 3), which was consistent with the element distribution obtained by the EDS mapping (Figure 4). The liquid phase was an important factor for the morphology of the phase. In general, the formation of liquid likely promoted the crystallization and growth of grains due to the faster diffusion rate [19]. Thus, the formation of granular spinel was most likely related to a lower amount of liquid phase formation in sample MT2.

### 3.3. Physical and Mechanical Properties

**Figure 6:**
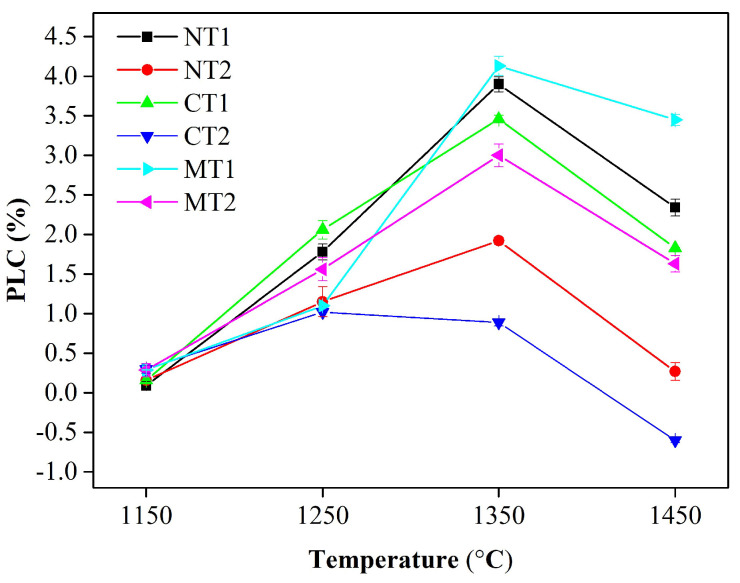
Permanent linear change (PLC) of specimens calcined at different temperatures.

PLC is an important parameter that represents the volume stability of refractories. As presented in Figure 6, the formation of spinel and CA_6_ caused volume expansions of 8 and 3% [10], respectively, which resulted in the continuous increase in PLC for all samples until 1350 °C, except for sample CT2. With the temperature further increasing, the shrinkage caused by sintering effects and phase evolution (liquid formation) dominated the calcination process [6], which resulted in a dramatic decrease in PLC for all samples above 1350 °C. Martinez [25] and Sako [18] stated that a liquid phase was first generated in the CaO-Al_2_O_3_-SiO_2_ ternary system, and its chemical composition was similar to that of gehlenite (C_2_AS), containing a small amount of Na_2_O in the matrix of alumina–magnesia castables. In addition, titania could assist the formation of liquid in the CaO-Al_2_O_3_-SiO_2_ ternary system [13]. It was demonstrated that liquid was generated in the local area where CaO, Al_2_O_3_, SiO_2_ and Na_2_O co-existed. As more titanium followed the calcium in the system described above, more liquid was generated. As mentioned above, the uneven distribution of titanium was designed by the incorporation of three different Ti-bearing compounds. It was assumed that titanium tended to distribute into calcium and magnesium in samples with CaTiO_3_ and MgTiO_3_ addition, respectively, and titanium was uniformly distributed in castables with nano-TiO_2_ addition. Thus, more liquid formed, and less volume expansion occurred for the samples with CaTiO_3_ and nano-TiO_2_ addition after calcination at 1350 and 1450 °C. In contrast, samples with MgTiO_3_ addition contained less liquid phase and exhibited greater volume expansion.

**Figure 7:**
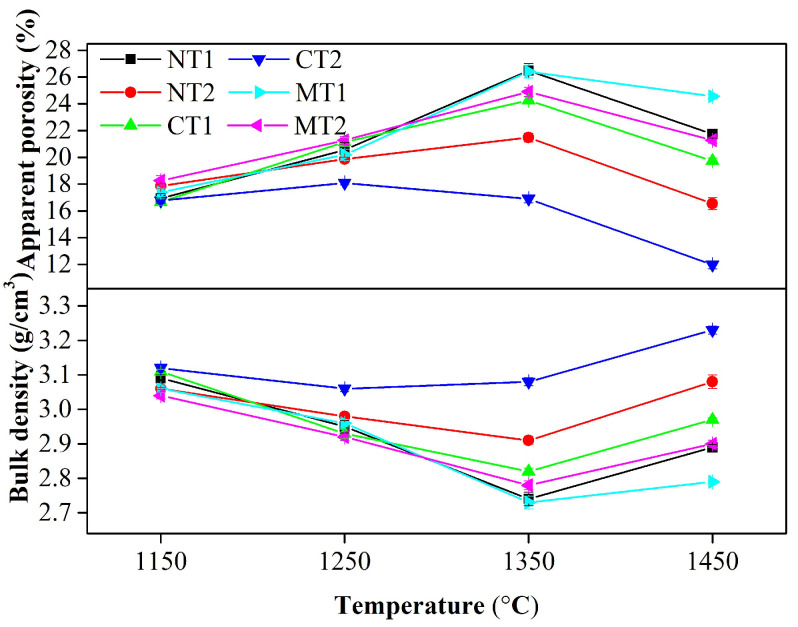
Apparent porosity and bulk density of samples after calcination at different temperatures.

The densification of castables was closely related to the thermal shock and slag resistances of the materials. The variations of the apparent porosity and bulk density of the samples as the temperature increased are presented in Figure 7. It was found that the variations of the apparent porosity of castables were consistent with those of the PLC of samples and were opposite to the variations of the bulk density. The apparent porosity of alumina-magnesia castables without mineralizer addition reached 23% after calcination at 1450 °C for 5 h [13]. For comparison, the apparent porosities of samples MT1 and MT2 were about 24% and 20%, respectively, which indicated that MgTiO_3_ had little effect on the densification of alumina-magnesia castables. However, a denser body of castables was obtained by the incorporation of two other compounds, especially CaTiO_3_.

**Figure 8:**
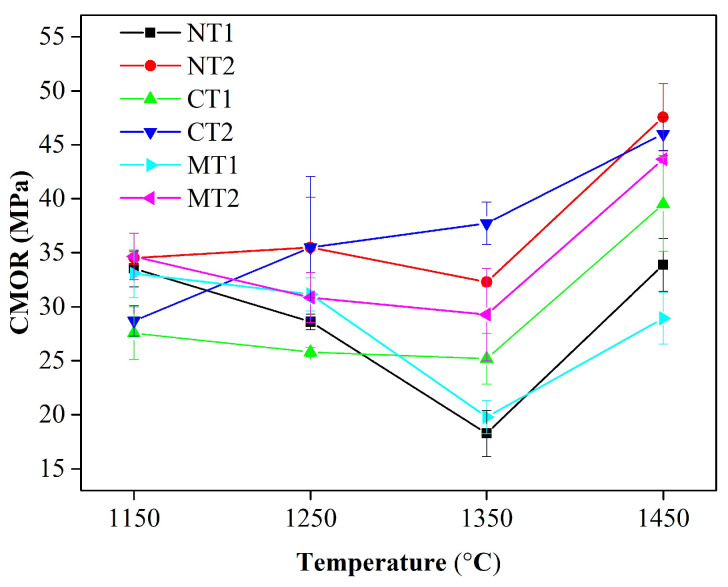
Variation of cold modulus of rupture for the castables with calcination temperatures.

Figure 8 presents the cold modulus of rupture (CMOR) values of the castables calcined at different temperatures. The strengths of the samples had the lowest values after calcination at 1350 °C, except for sample CT2, which underwent the largest volume expansion. In general, a higher bulk density produces a positive influence on the strengths of materials. Nevertheless, the strengths of samples MT2, NT2, and CT2 were similar after calcination at 1450 °C, while their bulk densities and apparent porosities were significantly different, as presented in Figure 7. When CA_6_ was interwoven and stacked, the strengths of the samples were enhanced [6,26]. The amounts of CA_6_ in samples CT2 and NT2 were less than that in sample MT2, as shown in Figure 3. Thinner, flaky CA_6_ was stacked in the matrix of sample MT2. However, thicker plates of CA_6_ were scattered in samples NT2 and CT2, as shown in Figure 5. The decrease in the CA_6_ content and its morphological changes hindered the further improvement of the strength.

**Figure 9:**
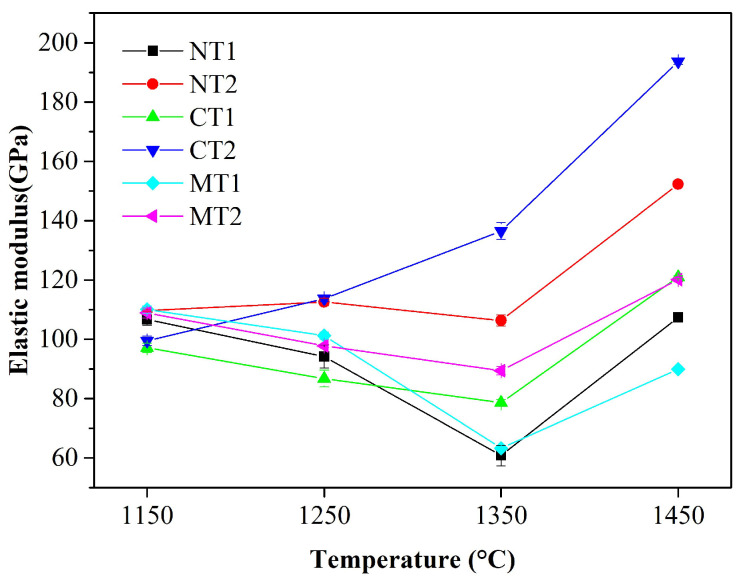
Variation of elastic modulus for the castables with calcination temperatures.

The evolution of the phase, texture, and apparent porosity have a major impact on the elastic modulus (E) of refractories [27,28]. The elastic modulus of the samples calcined at different temperatures were measured and are presented in Figure 9. The variations of the elastic modulus of the samples were the same as those of the bulk densities, which indicated the elastic modulus of alumina-magnesia castables strongly depended on the densification degree.

For practical applications, refractories need to undergo rapid temperature changes. High-strength refractories are much more susceptible to thermal shock damage [29]. The stored elastic strain energy at the stress level of σcan be quantified as σ^2^/E [30], which plays a critical role on the crack extension. A lower value of σ^2^/E corresponds to better thermal shock resistance. The values of σ^2^/E and the strengths and elastic modulus for samples calcined at 1450 °C for 3 h are listed in Table 4, where parameters σ and E represented CMOR and elastic modulus in this work, respectively. Although the σ^2^/E value of sample MT1 was lower than the others, this sample could not bear thermal stress, as it had the lowest strength. In general, there were no significant differences in the strengths of samples NT2, CT2, and MT2. The elastic modulus of sample CT2 was significantly higher than those of NT2 and MT2 because of the lower porosity. The resistance to thermal shock damage was inversely proportional to the elastic strain energy [31]. Thus, sample CT2, with a relative lower value of σ^2^/E, was estimated to have greater thermal shock resistance.

## 4. Conclusions

After comparing the physical properties of samples containing different Ti-bearing compounds (nano-TiO_2_, MgTiO_3_, and CaTiO_3_), the mechanisms through which titanium affected the phase and microstructural evolution were revealed. CaTiO_3_ formed first in alumina-magnesia castables containing TiO_2_ and MgTiO_3_ calcined at 1150 °C. As the calcination temperature increased, Ti gradually dissolved in CA_6_ and spinel. The influence of titanium on the solid-solution reaction of CA_6_ was more prominent than that of spinel. The thicknesses of the CA_6_ platelets increased as more titanium was incorporated into the crystal lattice. The enrichment of titanium with calcium promoted the formation of liquid. Therefore, the sintering of castables and the growth of spinel were greatly accelerated due to the faster transfer rate in liquid. Lower apparent porosities and greater strengths of alumina-magnesia castables were achieved by the addition of CaTiO_3_. Although castables with CaTiO_3_ and TiO_2_ additions had higher bulk densities than those of the castables with MgTiO_3_, their strengths were similar due to the decreasing contents and different morphologies of CA_6_. Based on the lower values of σ^2^/E, the castables containing 3.4 wt.% CaTiO_3_ (equivalent weight of 2 wt.% TiO_2_) were estimated to exhibit better thermal shock resistance.

## Figures and Tables

**Table 1:** Formulations of alumina-magnesia refractory castables.

Raw Materials	Content (wt.%)					
	NT1	NT2	CT1	CT2	MT1	MT2
Tabular alumina (d = 1–6 mm)	33.5	33.1	33.5	33.1	33.5	33.1
Tabular alumina (d = 0.2–1 mm)	27.5	26.9	27.5	26.9	27.5	16.9
Tabular alumina (d ≤ 0.074 mm)	15	15	15	15	15	15
Tabular alumina (d ≤ 0.045 mm)	3	3	4.6	6.3	3	3
Reactive alumina (CL370)	7	7	7	7	7	7
Calcined magnesia (d ≤ 88 μm)	6	6	6	6	5.5	5
Calcium aluminate cement (Secar71)	6	6	3.7	1.3	6	6
Silicon fume (951U)	1	1	1	1	1	1
Nano-TiO_2_	1	2	-	-	-	-
CaTiO_3_	-	-	1.7	3.4	-	-
MgTiO_3_	-	-	-	-	1.5	3

**Table 2:** Chemical compositions of raw materials.

Raw Materials	Chemical Compositions (wt.%)					
	Al_2_O_3_	MgO	SiO_2_	CaO	Na_2_O	Fe_2_O_3_
Tabular alumina	99.5	-	≤0.02	-	0.4	-
Reactive alumina (CL370)	99.7	-	0.03	0.02	0.1	0.03
Calcined magnesia (d ≤ 88 μm)	0.52	94.6	1.6	1.41	0.32	1.31
Calcium aluminate cement (Secar71)	≥68.5	<0.5	<0.8	≤31.0	<0.5	<0.4
Silicon fume (951U)	0.4	0.6	96.1	0.3	0.2	0.1

**Table 3:** EDS analysis of CA_6_ and spinel in different samples calcined at 1450 °C for 5 h (at. %).

Phase	Samples	Ca	Ti	Mg	Al	O
CA_6_	MT2	3.9	2.1	1.8	22.4	69.8
	NT2	3.9	3.0	2.2	31.5	59.4
	CT2	3.5	3.3	3.0	33.6	56.6
Spinel	MT2	0.4	0.2	11.8	26.9	60.7
	NT2	0.2	0.3	12.1	38.3	49.1
	CT2	0.3	0.3	12.8	29.3	57.3

**Table 4:** Results of strength, elastic modulus, and σ^2^/E of samples calcined at 1450 °C for 5 h.

Parameters	NT1	NT2	CT1	CT2	MT1	MT2
CMOR (MPa)	33.9	47.6	39.5	46.0	28.9	43.7
Elastic modulus (GPa)	107.4	152.3	121	193.7	89.9	120.1
σ^2^/E (kPa)	10.70	14.85	12.90	10.92	9.32	15.86

## Data Availability

The data are not publicly available.
